# Deep learning applied to analyze patterns from evaporated droplets of *Viscum album* extracts

**DOI:** 10.1038/s41598-022-19217-1

**Published:** 2022-09-12

**Authors:** Carlos Acuña, Alfonso Mier y Terán, Maria Olga Kokornaczyk, Stephan Baumgartner, Mario Castelán

**Affiliations:** 1 Robotics and Advanced Manufacturing, Center for Research and Advanced Studies of the National Polytechnic Institute, 25900 Ramos Arizpe, Mexico; 2 Society for Cancer Research, 4144 Arlesheim, Switzerland; 3 Institute of Integrative Medicine, University of Witten-Herdecke, 58313 Herdecke, Germany; 4 Institute of Integrative and Complementary Medicine, University of Bern, 3010 Bern, Switzerland

**Keywords:** Scientific data, Machine learning, Pharmaceutics

## Abstract

This paper introduces a deep learning based methodology for analyzing the self-assembled, fractal-like structures formed in evaporated droplets. To this end, an extensive image database of such structures of the plant extract *Viscum album Quercus* $10^{-3}$ was used, prepared by three different mixing procedures (turbulent, laminar, and diffusion based). The proposed pattern analysis approach is based on two stages: (1) automatic selection of patches that exhibit rich texture along the database; and (2) clustering of patches in accordance with prevalent texture by means of a Dense Convolutional Neural Network. The fractality of the patterns in each cluster is verified through Local Connected Fractal Dimension histograms. Experiments with Gray-Level Co-Occurrence matrices are performed to determine the benefit of the proposed approach in comparison with well established image analysis techniques. For the investigated plant extract, significant differences were found between the production modalities; whereas the patterns obtained by laminar flow showed the highest fractal structure, the patterns obtained by the application of turbulent mixture exhibited the lowest fractality. Our approach is the first to analyze, at the pure image level, the clustering properties of regions of interest within a database of evaporated droplets. This allows a greater description and differentiation of the patterns formed through different mixing procedures.

## Introduction

The generation of organized patterns of varying complexity as a result of fluid drying processes is a common but intriguing phenomenon in nature^1,2^. One possible way to obtain such patterns is the Droplet Evaporation Method (DEM), based on the self-assembly of particles suspended in fluids in nano and microstructures during evaporation. DEM has found application in several fields: inkjet printing^3^, manufacturing of new electronic and optical materials including thin films and coatings^4,5^, drug discovery^6^, as also quality analysis^2,7–10^ and medical diagnostics^1,11–13^. The method has also been successfully applied to exhibit coupled physical mechanisms in blood^14^, protein-protein interactions^15^ or to study the morphology associated with polymers^16^ and liquid crystals^17^. It has also demonstrated efficacy in differentiating the production modalities of pharmaceutical preparations, amongst others studying the impact of mechanical energy applied^7–9^. For the further development of DEM, research on pattern evaluation techniques that allow fast and objective evaluations of large data sets is of great importance. Such datasets should be designed for analyzing multi-variability for the understanding of DEM phenomena, i.e., data that are dependent on several related factors is desirable. In a recent work^1^ pattern formation in drying blood droplets presents evidence, based on statistical models, of a correlation between the level of physical exhaustion of a person and the pattern obtained. These results and the advances in the study of microscopic imaging using machine learning provide a gateway in the development of DEM patterns analysis.

Pattern formation in evaporating droplets is still not a fully understood process. It is known that the formation and positioning of the deposit depend largely on the evaporation induced phase transition and therefore on the flow dynamics that occur during drop evaporation^18^. The dynamics of these flows (e.g. contact line pinning, Marangoni flow)^19^, will depend on the droplet initial conditions (e.g. composition, relative humidity, temperature^20^, presence of microbubbles, spatial organization of molecules, concentration^21^, other characteristics of the fluid^2^). However, when the drops evaporate in the same environment, their drying process depends on the fluid’s internal characteristics. Therefore, even a slight difference may lead to changes in the resulting patterns^22^. Investigations show that droplet evaporation of different solutions and in different conditions may lead to a great variety of different complex patterns, such as patterns with ring structures^23,24^, dendritic patterns^25^, hexagons^26^, and fractals^2,24^. This led to works such as the one in^27^, which sought to model the patterns obtained by the DEM through fractal descriptors, as an effort to generate automatic classification methods to reduce visual evaluation in big databases. Some of these descriptors have limitations as they characterize, at some degree, the represented structure, i.e., very different images may have similar fractal properties. For this reason, works like^28^ use more than two descriptors, aiming at developing a more robust identifier of an image, or a family of images that have similar texture.

In the last few years, deep learning^29^ has emerged as a powerful tool in computer vision and has attracted considerable attention in biomedical image analysis, as it has proved to be effective in its ability to handle complex microscopic images^30^. One popular deep architecture is the convolutional neural network (CNN) which has obtained great success in various tasks of microscopic image analysis, such as detection, segmentation, and classification^30,31^. Given the images and the corresponding labels, a CNN model can be learned to generate hierarchical data representations, which can be used for clustering patterns that share visual features^32^. In a previous work^33^, a feedforward neural network (FNN) model was generated to identify possible adulteration, coagulation and spoilage of dairy milk by generating the model through learning from scratch. Also, a model based on Neural Architecture Search Network (NASNet)^34^ was used to infer information from the protein dried droplet patterns^35^. The CNN variant called Densely Connected Convolutional Networks (DenseNet) has also been used in microscopy image analysis with promising accuracy^36^. A significant benefit of DenseNet is that it requires fewer parameters and calculations. This is because each layer takes all previous feature maps as inputs, improving gradient descent and encouraging feature reuse^37^.

In this paper, we propose a deep learning approach for clustering of DEM patterns obtained from differently produced *Viscum album Quercus* $10^{-3}$ dilution variants^38^. Each variant was prepared by a series of three successive dilution steps of a *Viscum album Quercus* extract in water; after each dilution step, one of the following mixing procedures was performed: (i) laminar, (ii) turbulent and (iii) diffusion based control. For pattern evaluation, we propose a methodology based on Regions of Interest (ROIs) analysis through two stages. The first stage consists of automatic patch sampling using a full texture selection criterion; in the second stage, a clustering of patches that share similar features is carried out through the iterative application of DenseNet. This strategy allows to automatically identify and discriminate texture patterns present in the database of DEM images. Once the patches are clustered, we verify their fractal degree using Local Connected Fractal Dimension histograms (LCFD)^39^. Additionally, we perform clustering experiments using co-occurrence matrices in order to provide deeper insights about the benefit of our approach.

## Materials and methods

The use of plants in the present study complies with international institutional guidelines. *Viscum album Quercus* extract for the DEM experimentation was procured by ISCADOR AG (Arlesheim, Switzerland). *Viscum album* L. was harvested from *Quercus robur* growing in natural habitats in Switzerland (belonging to the Iscador AG, therefore no permission for harvesting was needed). The plants were identified by Mirio Grazi (Society for Cancer Research, Arlesheim, Switzerland). Voucher specimen (C.H. Quaresma 18.329) was deposited at the Herbarium of the Faculdade de Formação de Professores, Universidade Estadual do Rio de Janeiro, Brazil. *Viscum album* preparations are used in medicine amongst others as supportive treatment for cancer^40–42^.

The DEM image database here analyzed was obtained at the Society for Cancer Research in Arlesheim, Switzerland and will be later on referred to as the VAQ-database. For the dilutions a salt (1:1 NaCl and KCl) solution ($4 \times 10^{-8}$ g L$^{-1}$) in water purified according to Pharm. Eur.^43^ was used (the small amount of salts served for inducing the crystallization process in desiccating droplets). Each variant was obtained by diluting the extract three times (first in a ratio 1:20, and in the second and third step in a ratio 1:10); after each dilution step, 100 ml of the solution was placed in 250 ml Erlenmeyer flasks and mixed for 2 minutes and 30 seconds by inducing (1) turbulent flow (variant T; mixed by machine-made vertical, vigorous strokes) or (2) laminar flow (variant L; mixed by a series of successive vortex-like flows induced by hand in an up-side-down hold Erlenmeyer flask), or (3) by diffusion, as control (variant D; gently mixed by performing few very slow movements with a glass stirrer and left for ca. 30 min). The three variants were analyzed by means of DEM in experiments performed on 5 different days.

**Figure 1:**
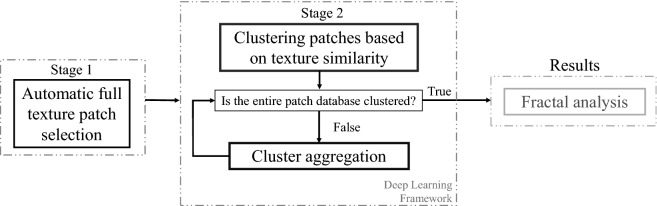
Flowchart of the proposed approach for automatic DEM texture analysis using deep learning. The texture analysis is based on two stages: (1) automatic selection of patches that observe rich texture along the database and (2) using DenseNet to determine clusters that present texture similarity. The fractality degree of each cluster is verified through LCFD histograms.

DEM images were obtained as described in^8^, in short: 2.5 $\upmu$l droplets of the *Viscum album Quercus* $10^{-3}$ variants (T, L, and D) were deposited on clean microscope slides and dried in a climatic cabinet, in controlled and constant conditions at 26 $^\circ C$ and 44$\%$ RH. After drying each droplet was photographed under a dark-filed microscope (Zeiss Lab.A1; Carl Zeiss Microscopy GmbH, Jena, Germany) with an attached camera (Moticam 5.0 MP; CMOS; Motic Electric Group Co., Ltd, Xiamen, China) in magnification 100$\times$ and saved in JPG file format. The VAQ database, which consists of 606 images of size 960 $\times$ 720 pixels (204 images of variant D, 206 of variant T, and 196 of variant L), was provided by the Society for Cancer Research under a data-sharing agreement. A background removal process was applied to all images using the Rolling Ball and Sliding Paraboloid method, in which a local background value is determined for every pixel by averaging over a very large ball around the pixel. This value is hereafter subtracted from the original image to remove large spatial variations in background intensities. The radius should be set to at least the size of the largest object that is not part of the background^44^. The result of this approach is an image almost free of glare effects, which is a consequence of the physical nature of microscopic images. It is important to note that the database does not present saturation or defocused conditions, as this would reduce the ability of the model to discriminate among textures. The proposed DEM texture analysis method is shown in the flowchart of Fig. 1. The remaining sections of the paper are organized in accordance with the flowchart as follows: first, we explain our method for automatic full-texture patch selection; the proposed strategy for patch clusterization based on texture similarity using deep learning is described later; finally, in order to verify the fractality degree of our results, we provide an analysis based on LCFD histograms as well as comparison with GLCM clustering results.

**Figure 2:**
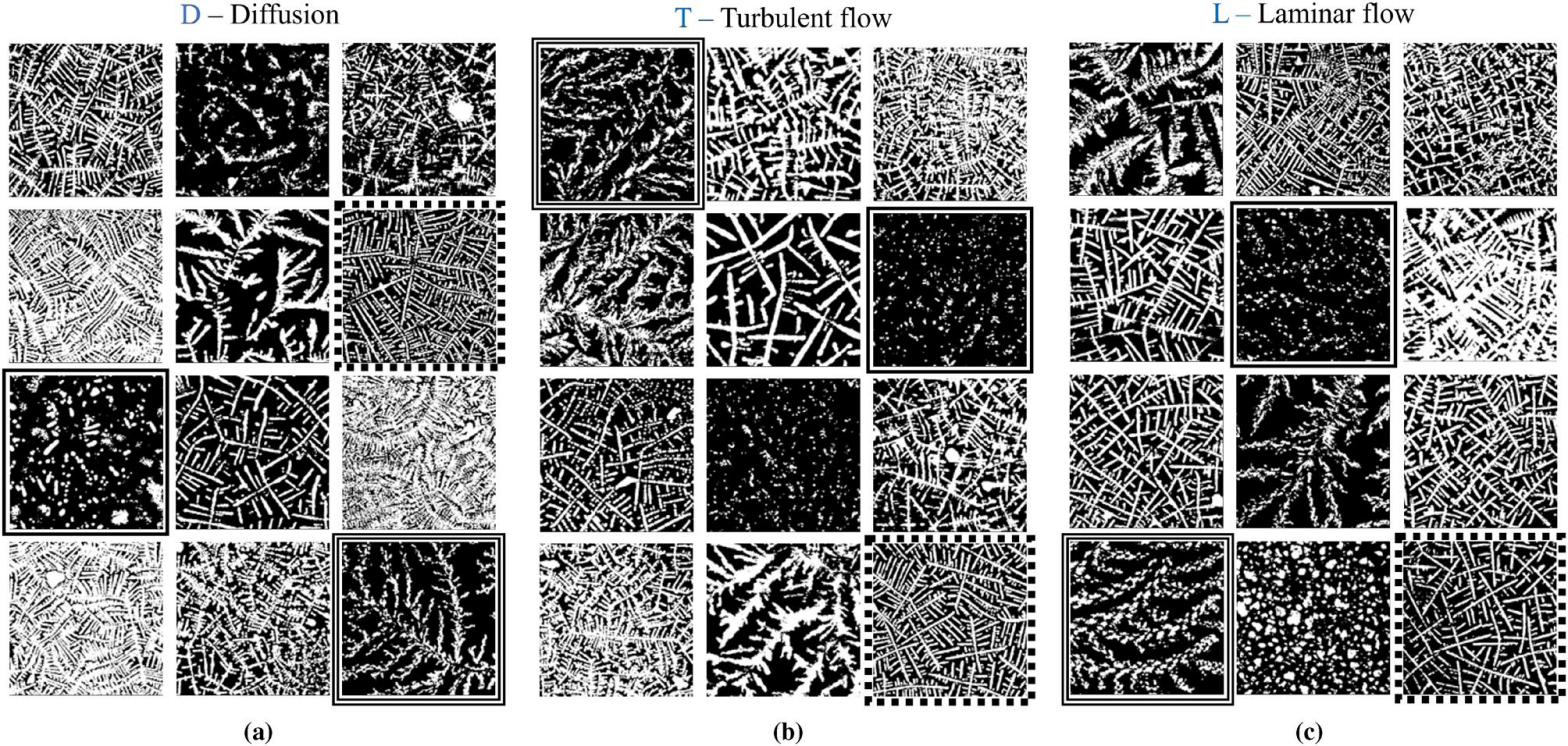
Texture diversity in some selected patches from different mixing procedures. The highlighted patches with dashed, solid and double frames from mixing procedure D share (visually) similar textures with the corresponding highlighted patches from mixing procedures T and L. (**a**) Patches from the D—diffusion control variant. (**b**) Patches from variant T—turbulent flow. (**c**) Patches from variant L—laminar flow.

## Stage 1: automatic full texture patch selection

The absence of substantial information in sections of the DEM image may be a problem when whole images are considered as inputs of CNNs. For the case of the VAQ database, not considering only regions of interest that exhibit complete texture would possibly lead to the learning of a bias by the network. The loss of resolution due to reducing the image size is also a factor to consider in order to benefit training time, GPU resources and performance of the model. These problems are illustrated in Fig. S1 of the supplementary information. To select the size of the patches to be used in our experiments, we borrowed ideas from the work carried out in^45^, in which the area under the receiver operating characteristic curve (ROC) was compared as a function of the input image resolution for DenseNet121^37^ and ResNet34^46^. In the cited work, it was shown that images of size 128 x 128 and 256 x 256 increase the performance of neural network for various machine learning tasks.

Motivating by the idea of preserving the resolution of the image so as not to lose valuable information about the texture, and with the aim of minimizing bias information, we propose to analyze the DEM database through image patches of size 128 $\times$ 128 that contain full texture information. To this end, we develop an automatic full texture patch selection. The patch selection stage begins with random sampling, which considers at most thirty percent of overlapping between patches, in order to reduce repetitive texture features up to approximately a 1:3 ratio, thus improving the quality of the patch database in the context of CNN training. An overall scheme of the automatic full texture patch selection process is illustrated in Fig. S2 of the supplementary information. The evaluation of the texture distribution in the patch is carried out by skewness analysis PCA for outlier removal, described below.

### Analysis of the pixel distribution

After random patch sampling, skewness analysis is performed to measure the lopsidedness of the pixel distribution. We compare the mean asymmetry of each row and column to detect the absence of texture information in the patch. When there is uniform pixel distribution in rows and columns (due to the symmetric behavior of texture information) we have a full texture patch, some of which are shown in Fig. 2. Patches that present absence of texture information (bias in texture location) exhibit high and/or low values in rows and columns. This effect is depicted in Fig. S3 of the supplementary information.

In order to preserve only complete texture patches, we evaluate two parameters of both distributions generated through the mean skewness values of each row and column:

- The first parameter is the standard deviation of the distributions, which is compared with a threshold to limit the dispersion of the pixels in the selected patches.
- The second parameter is the slope of the lines obtained from a first-order polynomial fit of the distributions.

We set 0.95 as a threshold for the standard deviation and 0.51 degrees for the slopes, since the patches with values less than these thresholds preserve a uniform pixel distribution. After skewness analysis is performed, and to improve bias reduction in the pixel distribution of the selected patches, outlier removal using patch-based PCA is carried out. The procedure is illustrated in Fig. S4 of the supplementary information. As a consequence of the automatic full texture patch selection (Stage 1), the final patch database consists of 1086 patches from the D—diffusion control variant, 705 from variant T—turbulent flow and 974 from variant L—laminar flow.

Through the visual examination of the obtained patches it is possible to see how the different mixing procedures appear to share similar textural patterns, since they come from the same plant extract; Fig. 2 provides examples of this: the highlighted patches with dashed, solid and double frames from mixing procedure D share a similar texture with the corresponding highlighted patches from mixing procedures T and L. As human visual inspection may not necessarily be objective, machine learning or other statistical methods are useful to corroborate human claims based on visual criteria. In this article, we determined the predominant texture pattern in each mixing procedure by clustering patches that exhibit texture similarity using DenseNet. The clustering process corresponds to the second stage of the proposed DEM texture analysis method and is described in the following section.

**Figure 3:**
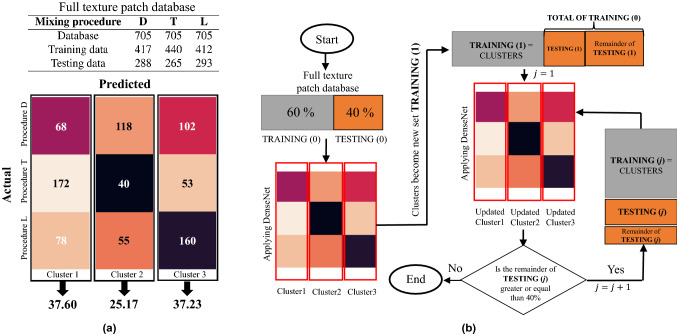
The proposed patch clustering method. (**a**) First clustering using DenseNet121. In the confusion matrix, *Actual* corresponds to the mixing procedures and *Predicted* to the clusters. (**b**) Flowchart of the iterative cluster aggregation process applied the whole patch database.

## Stage 2: patch clustering based on texture similarity using deep learning

In order to reduce learning bias, we trained DenseNet121 with the same number of samples per category. To this end, 705 patches were randomly selected from each mixing procedure (60 percent for training and 40 percent for testing), since this number was the maximum quantity of patches selected from the T mixing procedure. Figure 3a (top) shows the number of patches used for the first clustering. It is important to note that, during the batch normalization process, DenseNet121 defines non-trainable parameters; for this reason, the final number of training patches were not equally distributed.

Results of the first clustering are presented in Fig. 3a (bottom) with the help of a confusion matrix, where *Actual* is the original clusters formed by the mixing procedures D, T, and L and *Predicted* is the new clusters formed by the procedures D, T, and L through DenseNet121. Note how the columns of the matrix represent what the network has learned as similar textures. The matrix shows that 37.6 percent of the test patches were categorized in *Cluster 1*, 25.17 percent in *Cluster 2* and the remaining 37.23 percent in *Cluster 3*. In this initial step, 40$\%$ of the full texture patch database was clustered based on texture similarity and 60$\%$ was used for training. In order to categorize the patches used for training into the clusters defined by DenseNet121 in the initial clustering, we propose the iterative algorithm shown in Fig. 3b. The inputs to the algorithm are the subsets of patches used for initial testing and training, defined as TESTING(0) and TRAINING(0), respectively. In the first iteration ($j=1$), the clustered patches TESTING(0) become the new training subset TRAINING(1) and the non-clustered patches TRAINING(0) become the new testing subsets. DenseNet121 is then applied to TRAINING(1) and TESTING(1), which should maintain a 60:40 ratio. The necessary condition for further iterations (applications of DenseNet121) is that the Remainder of TESTING(j) must be greater than or equal to 40$\%$ compared to the sum of TRAINING(j) and TESTING(j).

**Figure 4:**
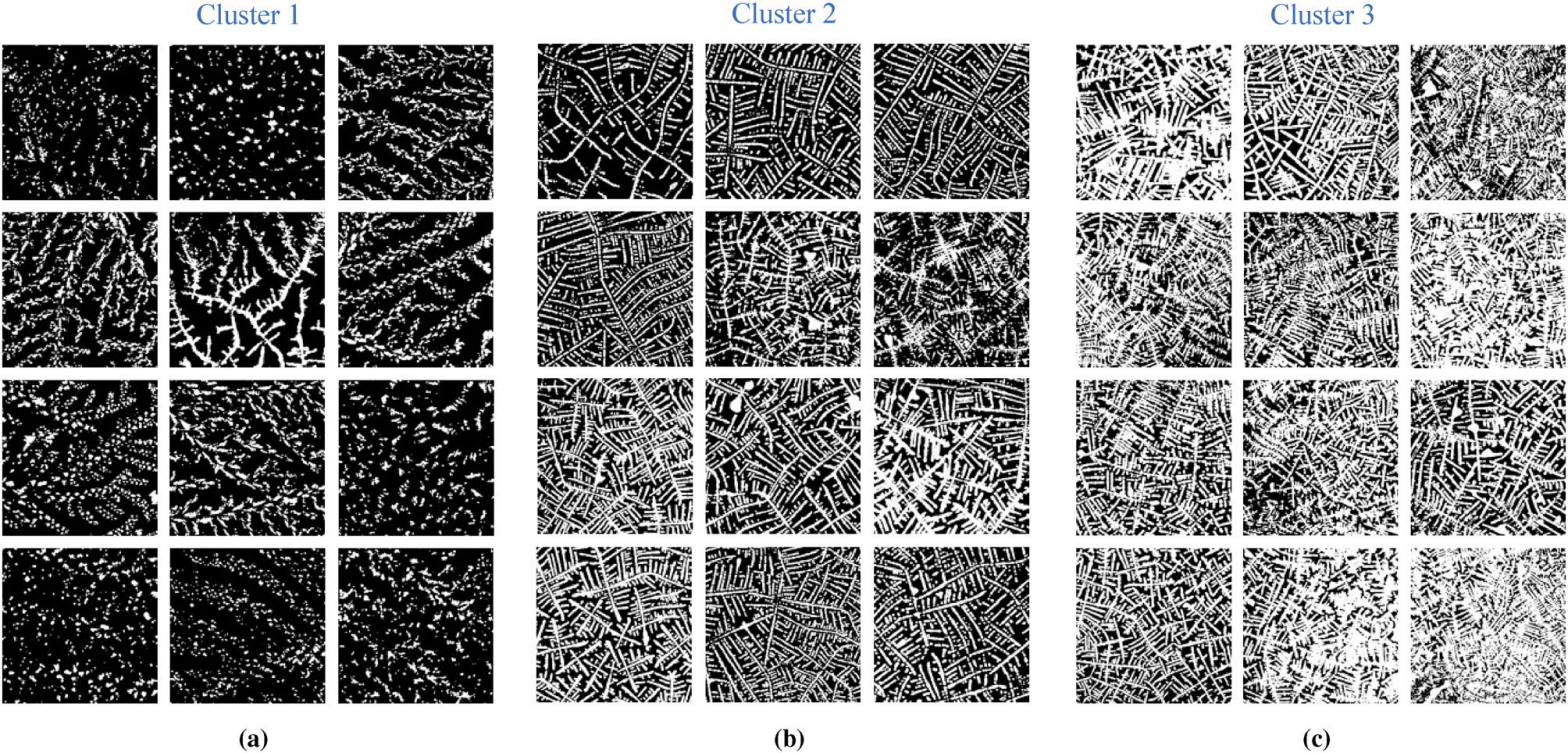
Examples of patches resulting after DenseNet based clusterization. (*a*) patches from cluster 1. (*b*) patches from cluster 2. (*c*) patches from cluster 3.

## Results

After having applied the patch clustering process described in the previous section, the noticeable visual differences between the clustered texture patterns can be analyzed in Fig. 4, where examples of patches of the three formed clusters reveal gradual connectivity among pixels, resembling fractality. As far as the number of clustered patches is concerned, Cluster 1 had the greatest participation in the DEM pattern images with 782 patches, followed by Cluster 3 with 740 patches, and finally, Cluster 2 with 593 patches. Note how the texture patterns of Cluster 1 exhibit the greatest particle scattering, contrary to Cluster 3, which exhibits the greatest particle connectivity. The texture patterns of Cluster 2 also present particle connectivity, but lower, compared to Cluster 3.

In order to verify the degree of fractality in the clustered textures, a fractal evaluation of each cluster was performed. To this end, LCFD histograms were obtained from each of the patches in the database before and after applying the proposed clustering method. In addition, we applied patch texture clustering based on properties obtained through gray level co-occurrence matrices (GLCM)^15^. For the latter, a PCA was constructed from a vector ${\textbf {t}}$ of size $(m \times n) \times 1$, where $m=4$ are the contrast, correlation, energy and homogeneity features extracted from each of the GLCM matrices and $n=4$ are the four matrices obtained from $0^\circ$, $45^\circ$, $90^\circ$ and $135^\circ$ offsets. The three clusters were calculated in the projection space of the first three principal components using the agglomerative hierarchical clustering algorithm^47^.

**Figure 5:**
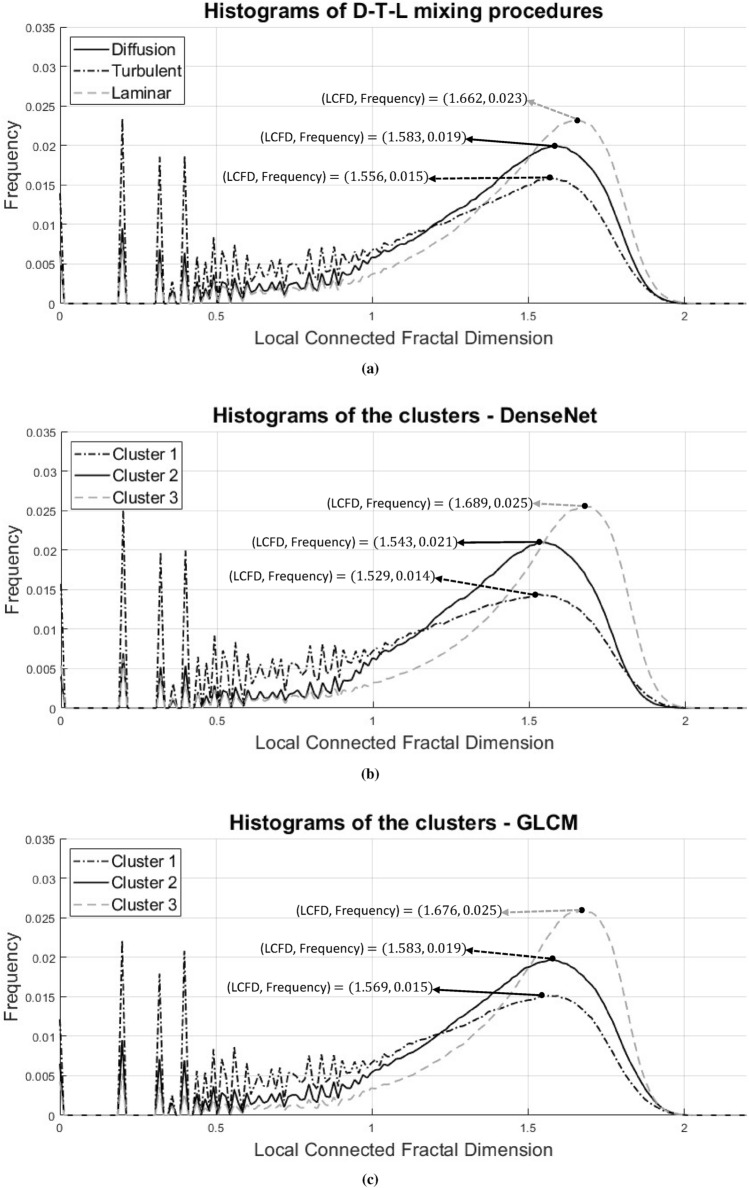
Average LCFD histograms of the full texture patch database. (**a**) Average LCFD histograms of the D, T and L mixing procedures. (**b**) Average LCFD histograms of the clusters determined by deep learning approach. (**c**) Average LCFD histograms of the GLCM clusters.

Figure 5a shows the average LCFD histograms of the 705 patches for each mixing procedure. Differences between histograms can be observed as a greater variation in the initial values of the T histogram and small differences in the LCFD and frequency values at the highest point of the bell curves formed in the distribution. This may be caused by the texture diversity presented in each mixing procedure (as previously depicted in Fig. 2). As far as our proposed method is concerned, each cluster is composed of patches that share similar texture features, thus their average LCFD histograms show greater separability compared to the mixing procedures histograms. This can be observed through the highest points of the bell curves formed in the distributions of Fig. 5b. For comparison, the average LCFD histograms of the GLCM clusters are shown in Fig. 5c. The plots reveal that GLCM Cluster 3 is the one that exhibits the highest frequencies for high LCFD values. GLCM Clusters 1 and 2 show similarity between their distributions since they present small variations between their LCFD and frequency values. It is worth noticing that, in accordance with Fig. 5b, Cluster 3 obtained through our method presents the highest fractality due to its large concentration of high LCFD values. The lowest fractality is exhibited by Cluster 1 of our approach, since its average histogram shows high frequencies for small LCFD values, and the lowest peak at LCFD values greater than 1.

**Table 1:** Results of the LCFD histograms for clusters. *CI* confidence interval 95$\%$.

LCFD—GLCM						LCFD—DenseNet					
Clusters	N	Mean	SE	Kurtosis	Skewness	Clusters	N	Mean	SE	Kurtosis	Skewness
Cluster 1	719	1.21	0.64	− 0.21	0.92	Cluster 1	782	1.18	1.16	− 0.30	0.95
Cluster 2	682	1.36	0.48	1.70	1.26	Cluster 2	593	1.37	0.78	2.16	1.34
Cluster 3	714	1.47	0.38	3.96	1.77	Cluster 3	740	1.48	0.74	3.71	1.76

*N* number of evaluated patches, *SE* standard error.

Statistical results that describe each LCFD histogram are presented in Table 1. These results are used to categorize each cluster according to its fractal degree. Considering the mean, standard error, kurtosis and skewness values, clusters for both GLCM and DenseNet can be categorized from less to more fractal, Cluster 3 being the one that presents the highest fractal degree, followed by Cluster 2 and Cluster 1. Note how for the case of GLCM, the kurtosis values reveal that the fractal degree of the first two clusters (i.e., less and medium fractal) is more similar (1.91 kurtosis distance) than their DenseNet counterparts (2.46 kurtosis distance), suggesting greater difference between the clusters obtained through the latter.

Aiming to provide statistical significance to the data described above, the separability of the different LCFD distributions was evaluated using the Kolmogorov–Smirnov two-sample nonparametric test (K–S test)^48^, due to its ability to differentiate between the shape and skewness of distributions where outliers and bias are present. The test was performed at a significance level of 0.05, that is, if the p-value was above 0.05, the test would indicate that both distributions are similar, i. e. the test would have a result of 0. The results shared in Table 2 indicate that only the distributions from the mixing procedures T and L are differentiable; distributions between mixing procedures D and L present the highest *p*-value, thus no distribution separability can be appointed between these modalities. In the case of GLCM, Clusters 1 and 3 (the least and most fractal ones) are highly differentiable, since they have *p*-values very close to zero, while the *p*-values for the distributions from Clusters 2–3 (the medium and most fractal ones) and the distributions from Clusters 1–2 (the least and medium fractal ones) are above the significance level, indicating that clusters whose fractality is next to each other are indeed similar. On the contrary, only the deep learning approach demonstrates separability between Clusters 1–2 and Clusters 1–3, having *p*-values close to zero. The *p*-values of the distributions of Clusters 2–3, however, are above the significance level (*p*-value of 0.14), suggesting a closer similarity between distributions of the medium and more fractal clusters. It is also to note that, in all cases, our proposed methodology achieves the smallest *p*-values.

**Table 2:** K–S test results of the LCFD distributions at the 5$\%$ significance level. P-values highlighted in boldface indicate distribution separability.

LCFD—Original				LCFD—DenseNet				LCFD—GLCM			
Hypothesis	T	D	L	Hypothesis	$C_1$	$C_2$	$C_3$	Hypothesis	$C_1$	$C_2$	$C_3$
T	0	0	**1**	$C_1$	0	**1**	**1**	$C_1$	0	0	**1**
D	0	0	0	$C_2$	**1**	0	0	$C_2$	0	0	0
L	**1**	0	0	$C_3$	**1**	0	0	$C_3$	**1**	0	0

LCFD—Original				LCFD—DenseNet				LCFD—GLCM			
*p*-values	T	D	L	*p*-values	$C_1$	$C_2$	$C_3$	*p*-values	$C_1$	$C_2$	$C_3$
T	0	0.2204	**0.0332**	$C_1$	0	**0.0434**	**0.0076**	$C_1$	0	0.1796	**0.0103**
D	0.2204	0	0.6044	$C_2$	**0.0434**	0	0.1426	$C_2$	0.1796	0	0.1651
L	**0.0332**	0.6044	0	$C_3$	**0.0076**	0.1426	0	$C_3$	**0.0103**	0.1651	0

H = 1 indicates that K–S test rejects the hypothesis.

**Figure 6:**
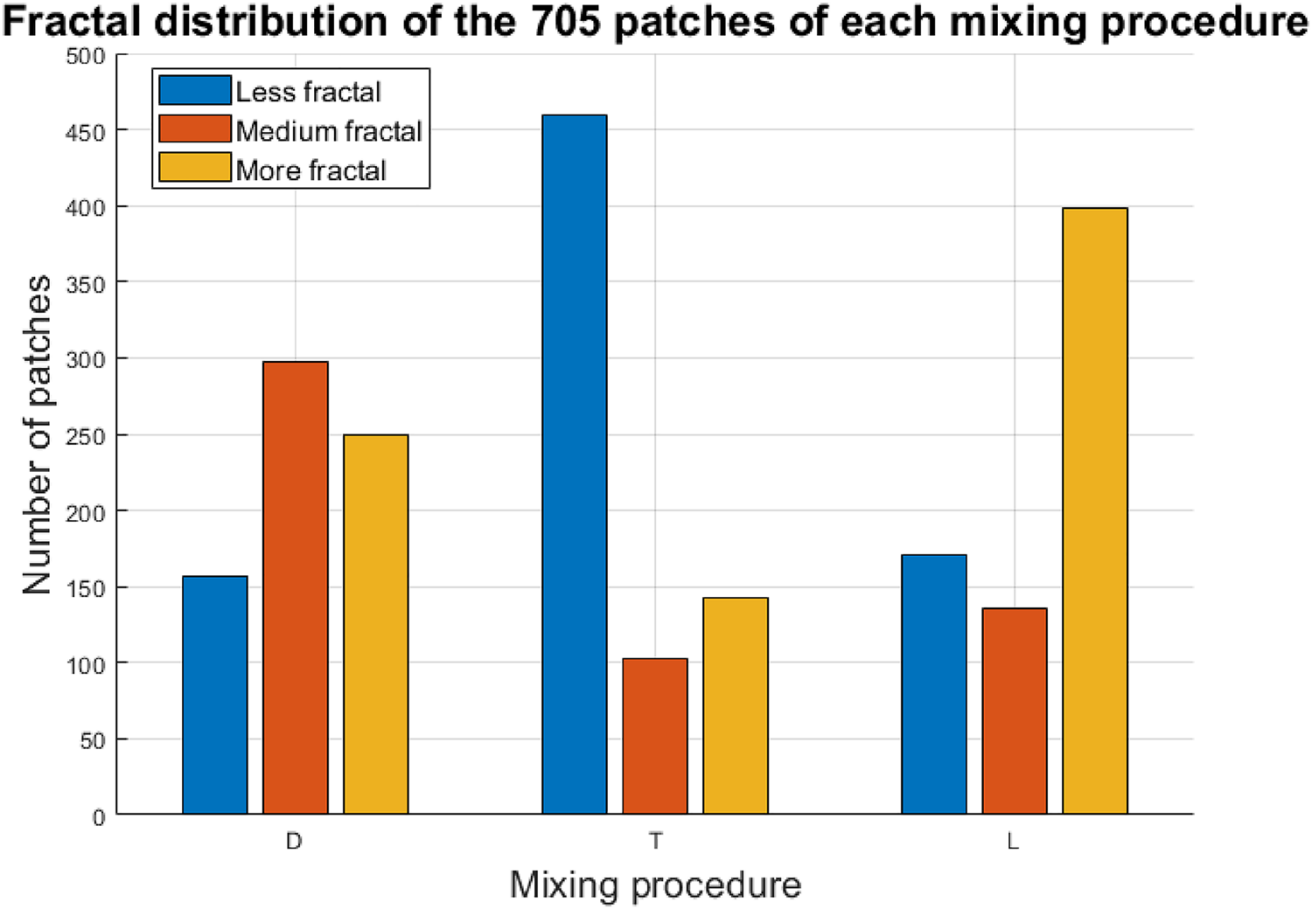
Fractal distribution of the mixing procedures. The patches obtained from the L mixing procedure (laminar flow) show the highest fractal composition, having 56.60 percent of the patches in the *More fractal* category. The patches obtained from the T mixing procedure (turbulent flow) exhibit the lowest fractal composition, having 65.2 percent of the patches in the *Less fractal* category.

The final categorization of clusters in terms of mixing procedures is depicted in Fig. 6. By categorizing each patch according to its degree of fractality, the following results were obtained: for the D mixing procedure, 22.2$\%$ of the 705 patches correspond to the category of less fractal, 42.2$\%$ to medium fractal, and 35.4$\%$ to more fractal; for the T mixing procedure, 65.2$\%$ of the 705 patches were categorized as less fractal, 14.6$\%$ as medium fractal, and 20.2$\%$ as more fractal; finally, for the L mixing procedure, 24.2$\%$ of the 705 patches were categorized as less fractal, 19.2$\%$ as medium fractal and 56.6$\%$ as more fractal. From the figure, it is to note that the proposed methodology for automatic DEM texture analysis allowed determining the fractal distribution of textures in the database for each mixing procedure. The results showed that the DEM patterns of the plant extract *Viscum album Quercus* $10^{-3}$ present the highest degree of fractality when mixed by laminar flow (L mixing procedure). The lowest degree of fractality is presented when mixed through the application of turbulent flow (T mixing procedure). Finally, most of the DEM patterns from the diffusion mixing procedure exhibit textures in the medium fractal category. In addition to determining the fractal composition of each mixing procedure, our method obtains the spatial location of the patches in the image. Examples of this application are shown in Figs. S5, S6 and S7 of the supplementary information.

## Discussion

Determining and exposing the variations in texture patterns that exist between the different pharmaceutical preparations of *Viscum album Quercus* $10^{-3}$ is a highly challenging problem for several reasons. First, the microstructure is particularly complex. Second, numerous microscopic images contain observable contaminations. Glare and visual pollution can be formed during sample preparation or image acquisition and result in an obstacle during the process of texture analysis. Third, variants prepared by means of the different mixing procedures share a great diversity of textures. Therefore, the use of traditional techniques such as gray level co-occurrence matrix (GLCM)^49–51^, and fractal-based measures^2^ can lead to ambiguities in texture differentiation. We have observed that LCFD histograms of DEM patterns are not unique. They characterize at some degree the represented structure, though completely different images may have similar LCFD histograms. In this context, deep learning techniques have provided better performance in the analysis of microscopic images^30^. For these reasons, the proposed methodology for automatic DEM texture analysis developed in this paper uses a Dense Convolutional Network (DenseNet), which allows texture patterns to be clustered based on their similarity.

It is known that the induction of different types of fluid movements may influence the properties of pharmacologically active compounds in solution. Turbulent flow may cause, *inter alia*, oxidation, aggregation of proteins^52^, formation of micro-bubbles^53^ and micro-particles^54^. In case of pharmaceutical preparations, this may lead to a decrease of the compound’s therapeutic potential. In a previous study, impact of turbulent flows could be associated with a decrease of DEM pattern complexity^8^. On the other hand, smooth fluid movement, like laminar flow, have been shown to trigger assembly of proteins into hierarchical macroscopic structures^55^, introducing so a new order to the solution. Interestingly, both kinds of flows, turbulent and laminar, are used in pharmaceutical manufacturing (e.g. for homeopathic preparations).

The results of the present study show that the three analyzed mixing procedures of the plant extract *Viscum album Quercus* $10^{-3}$ significantly influenced the DEM patterns. It can be summarized that mixing by turbulent flow induced the formation of structures characterized by smaller complexity (parameter LCFD), at the same time increasing the gaps between the structural elements, whereas the structures formed in samples mixed by laminar flow showed the greatest connectivity, and highest complexity. The DEM images of diffusion based mixed extracts present similar fractal characteristics as the L mixing procedure, however, the proposed method can differentiate slight differences between these two variants.

## Conclusions

In this paper, we have proposed an automatic pattern texture analysis of dried droplets of the plant extract *Viscum album Quercus* $10^{-3}$. The proposed method consists of two stages:

- A database of full texture patches from the DEM imagery is generated.
- Three clusters are defined according to the texture similarity of the patch database through a Dense Convolutional Network (DenseNet).
- The fractality degree of the clusters is determined by evaluating the average LCFD histograms of the obtained clusters.

With this method, we were able to show that the type of mixing during dilution (turbulent vs. laminar vs. diffusion) of the plant extract *Viscum album Quercus* $10^{-3}$ significantly influenced the patterns formed during the droplet evaporation. The mixing based on laminar flow induced the highest degree of fractality, having 56.60 $\%$ of the corresponding patches in the category of highest fractality, whereas the category of lowest fractality was assigned to most patches obtained from images of the variant mechanically mixed by turbulent flow. In the latter variant the particles were scattered, and consequently, there was a higher frequency of small values of LCFD.

As resulted out of three recent review articles concerning DEM applied as an analytical tool in pharmaceutics^10^ and medical diagnostics^11,56^ the main shortcoming and limitation of the method is the often exclusively applied visual pattern evaluation that can be subjective and thus a possible source of bias. The development and implementation of computerized image evaluation techniques that can be used complementary or alternatively to the visual evaluation is therefore of great importance for ensuring an objective DEM pattern evaluation.

In particular, an automatic pattern evaluation based on deep learning might contribute to further development of different applications of the droplet evaporation method by providing complementary image evaluation tools ensuring besides the result objectivity also time-saving evaluation procedures for large image databases.

## Supplementary Information

Supplementary Information.

## Data Availability

Correspondence and requests for programming codes and database should be addressed to M.C.
