# Supplementary material for: Deep learning applied to analyze patterns from evaporated droplets of Viscum album extracts
Source: Sci Rep. 2022 Sep 12;12:15332. doi: 10.1038/s41598-022-19217-1 (PMC9468023; doi:10.1038/s41598-022-19217-1)
Supplement: Supplementary file 1 — Supplementary Information. [file 41598_2022_19217_MOESM1_ESM.pdf]

Deep Learning Applied to Analyze Patterns from Evaporated Droplets of *Viscum album* Extracts

Carlos Acuña[1], Alfonso Mier y Terán[1], Maria Olga Kokornaczyk[2], Stephan Baumgartner[2,3,4], and Mario Castelán[1,*]

[1]Robotics and Advanced Manufacturing, Center for Research and Advanced Studies of the National Polytechnic Institute, Ramos Arizpe, 25900, Mexico
[2]Society for Cancer Research, Arlesheim, 4144, Switzerland
[3]Institute of Integrative Medicine, University of Witten-Herdecke, Herdecke, 58313, Germany
[4]Institute of Integrative and Complementary Medicine, University of Bern, Bern, 3010, Switzerland
[*]mario.castelan@cinvestav.edu.mx

Supplementary Information

Automatic full texture patch selection

In Figure S1, the absence of texture information in several sections of the DEM image is noticeable. The loss of resolution in the reduced DEM images is also exhibited in the figure. These represent common problems that appear when considering the analysis of the complete image using convolutional neural networks. For this reason, we turned our attention to texture information provided by image patches.

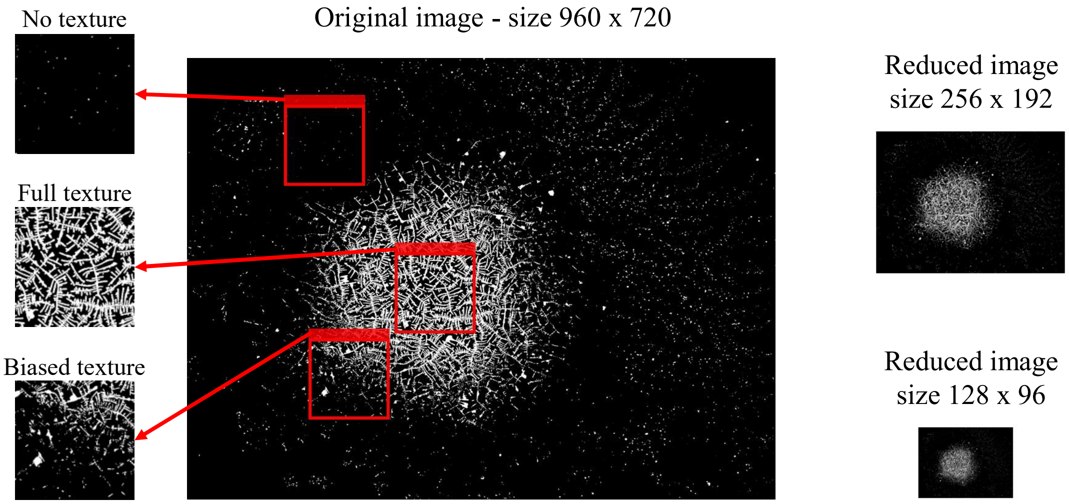

Figure S 1. Bias in texture patches and the scaling issue in the context of DEM images. The size perspective of the original DEM image is shown in the left part of the figure, highlighting sections with and without texture information, some of them exhibiting bias with respect to the localization of the textural pattern. Re-scaled versions of the original images are shown at the right part of the figure, exhibiting low resolution, which is not desirable for deep learning frameworks.

An overall scheme of the automatic full texture patch selection process is illustrated in Figure S2. The selection begins with the whole DEM image (Figure S2a), and proceeds with a random patch sampling (Figure S2b) considering at most thirty percent overlapping area between patches. The idea behind this is to reduce repetitive texture features between patches. The final selection is carried out by means of skewness analysis and PCA-based outliers removal. In Figure S2c, the resulting selected patches for the input image are depicted.

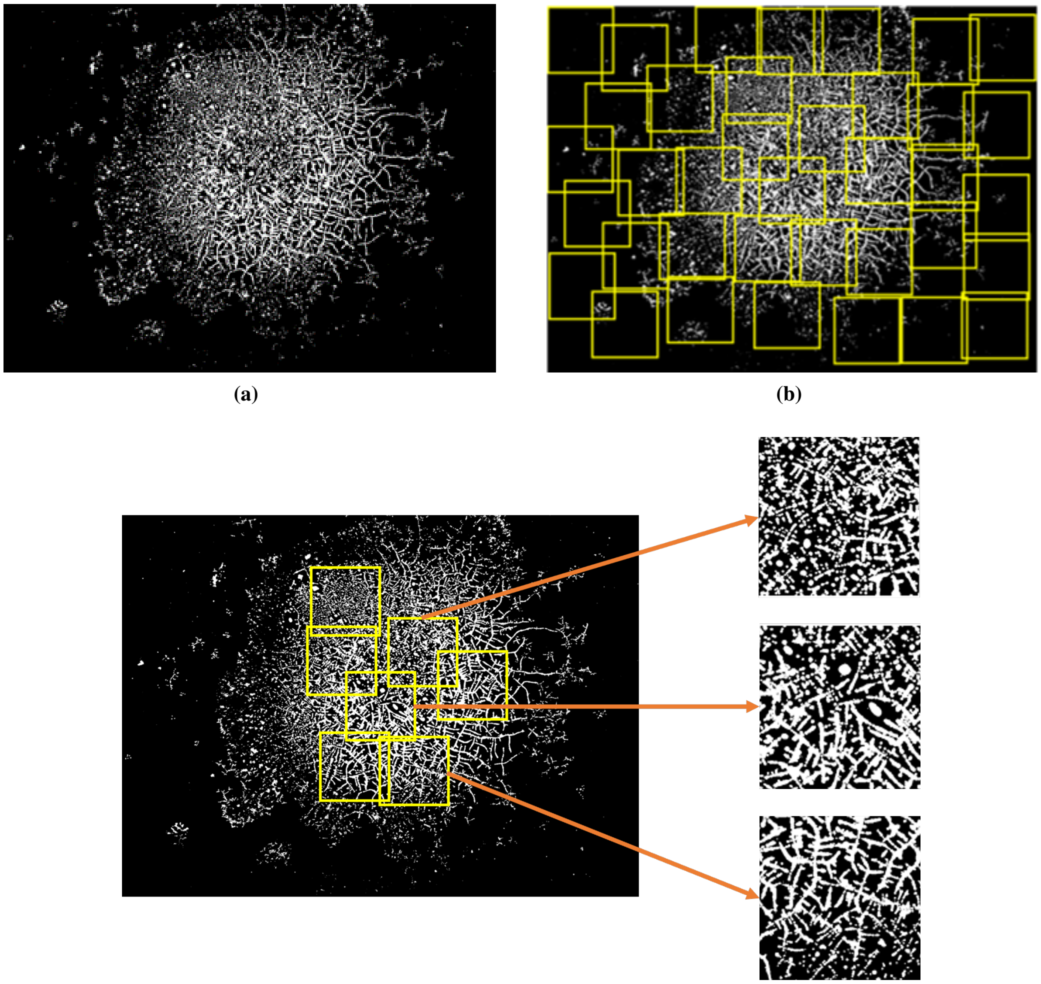

(a) (b) (c)

Figure S 2. Patch sampling process. (a) DEM image. (b) Random patch sampling, considering at most 30 percent of overlapping area. (c) Final patch selection after applying skewness analysis and PCA-based outliers removal.

Skewness analysis

Here we provide specifics about the skweness analysis, an important stage of the automatic selection of full texture patches. Once the random patch sampling has been performed, we measure the lopsidedness of the pixel distribution. An example of this for a couple of patches is shown in Figure S3. We compare the mean asymmetry measure of each row and column of the patch in order to detect the absence of texture information. Figure S3 (top) shows a patch that presents a uniform pixel distribution due to the symmetric behavior of its texture information. On the contrary, the patch shown in the bottom row of the figure exhibits absence of texture information, thus asymmetric behavior. The parameters that are evaluated to consider a complete texture patch are the standard deviation for both distributions generated through the mean values of skewness and the slopes of their first-order polynomial fit.

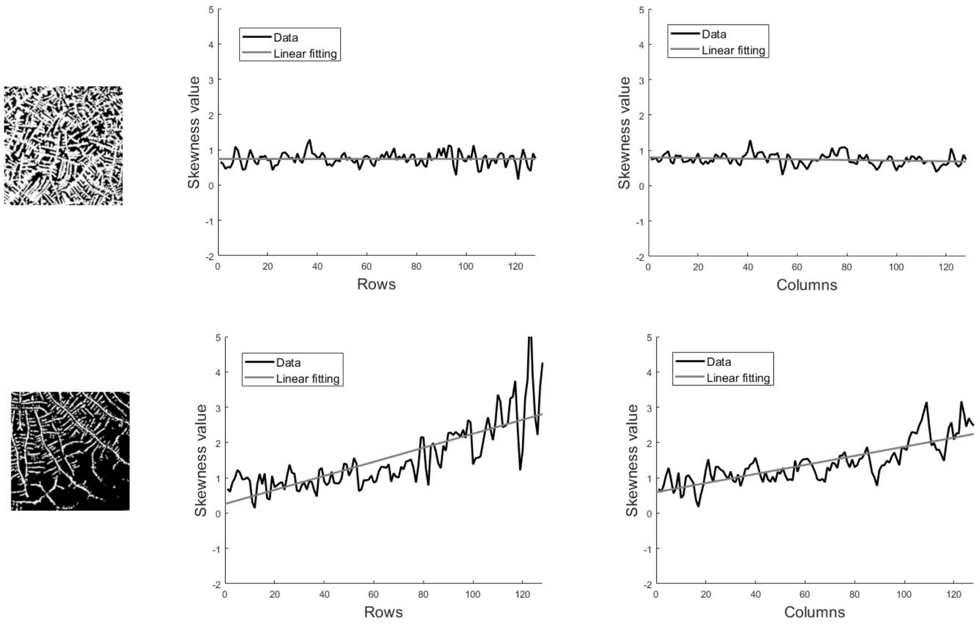

Figure S 3. Skewness analysis of the pixel distribution in the patches. (a) a uniform distribution in rows and columns is shown due to the symmetry of the pixels. (b) The skewness analysis shows high positive values in rows and columns due to the absence of texture information.

PCA for outlier removal process

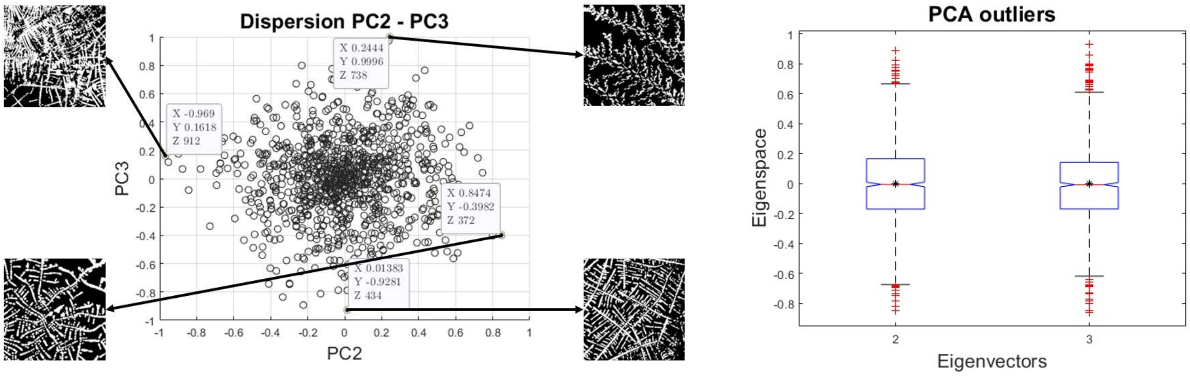

Figure S 4. Dispersion of the second and third eigenvectors. Left: the extreme values along the axis (outliers) correspond to patches that do not exhibit full texture. Right: a box plot is used to remove such outliers (shown with a red plus sign).

Here we provide specifics about the outlier removal process based on PCA, the final stage for the automatic selection of full texture patches. For PCA, each patch is transformed into a column vector $\mathbf{t}_{n\times 1}$, where $n = 128 \times 128$ is the number of pixels in the patch. The training set data matrix is constructed by concatenating each vector patch by columns as $[\mathbf{t}_1 \mid \mathbf{t}_2 \mid \cdots \mid \mathbf{t}_k]$, where k is the number of patches selected in the analysis of the pixel distribution. The differences from the patch average $\bar{\mathbf{t}}$ are used to construct the centered training data matrix $\mathbf{T}_{n\times k} = [(\mathbf{t}_1 - \bar{\mathbf{t}}) \mid (\mathbf{t}_2 - \bar{\mathbf{t}}) \mid \cdots \mid (\mathbf{t}_k - \bar{\mathbf{t}})]$.

Principal Component Analysis seeks a set of orthogonal vectors which, in the least square sense, minimize the correlation between the columns of $\mathbf{T}$. The solution is found by calculating the eigenvectors of the covariance matrix $\mathbf{\Sigma}_{n\times n} = \mathbf{T}\mathbf{T}^T$. As $\mathbf{\Sigma}$ is symmetric, there always exist and orthogonal basis $\mathbf{U}_{n\times n}$ and a diagonal matrix $\mathbf{\Lambda}_{n\times n}$ that satisfies the relationship:

$$\mathbf{\Sigma} = \mathbf{U}\mathbf{\Lambda}\mathbf{U}^T, \tag{1}$$

where $\mathbf{U}_{n\times n}$ is the eigenvector matrix and the eigenvalues of $\mathbf{\Sigma}$ are the diagonal elements of matrix $\mathbf{\Lambda}$.

We observed that the second and third principal components of the patch database were related to texture bias in the patches. Negative outliers for the second principal component presented a bias towards the upper right corner in the patches, while the bias for positive outliers was towards the lower left corner. For the third principal component, negative outliers showed a bias towards the upper left corner, while the bias for positive outliers was towards the lower right corner. These observations are depicted in Figure S4 (left). Finally, to obtain the full texture patch database, we removed the outliers from the second and third eigenvectors. These outliers are depicted with red plus signs in the boxplot of Figure S4 (left). Note that points are considered outliers if they are greater than $q_3 + w \times (q_3 - q_1)$ or less than $q_1 - w \times (q_3 - q_1)$, where w is the multiplier whisker, and q_1 and q_3 are the 25th and 75th percentiles of the sample data, respectively.

Complementary figures for the Results section

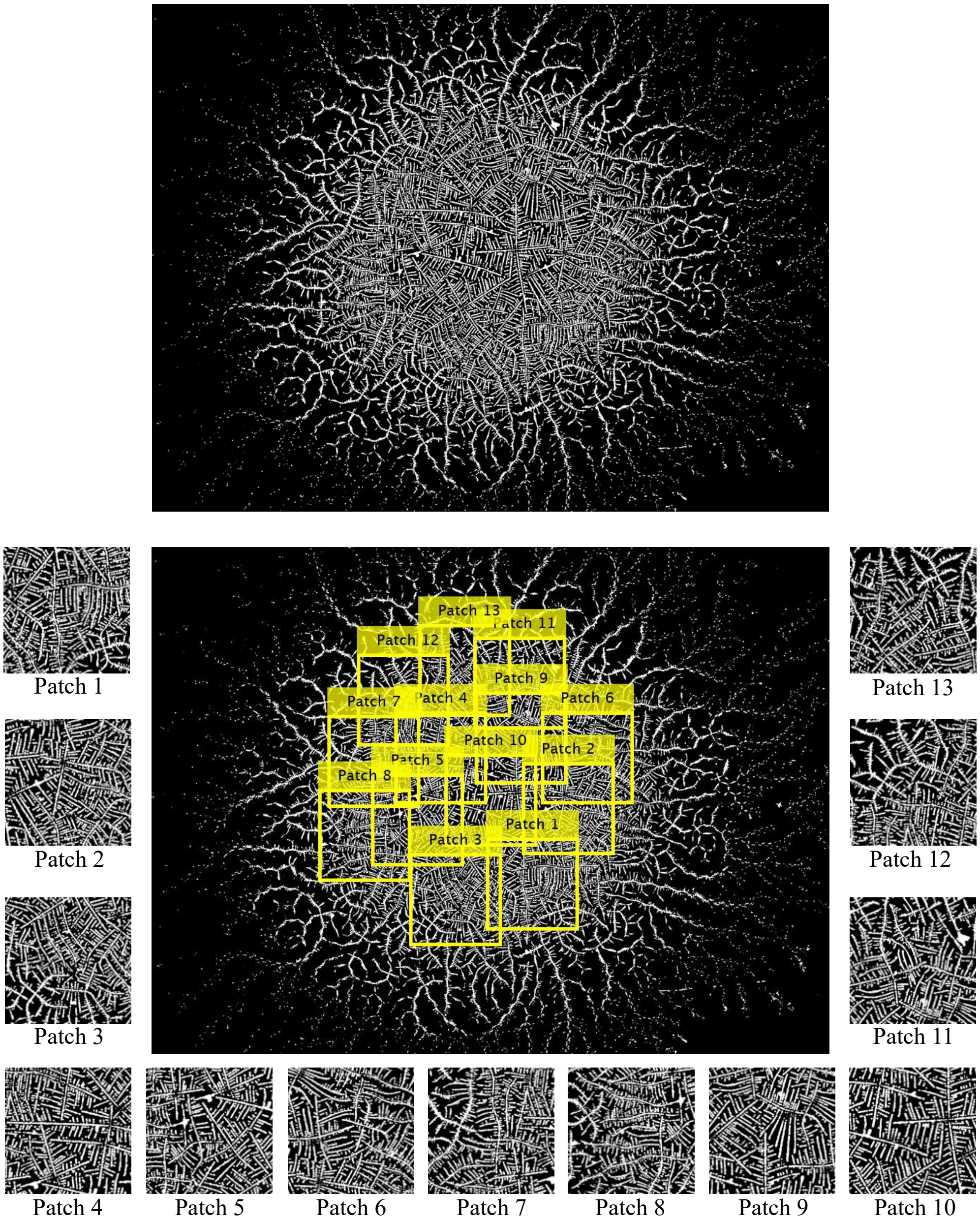

Figure S 5. D mixing procedure image and its spatial location of patches. Top: the image with the highest texture composition in the medium fractal category is shown. Bottom: the thirteen patches correspond to a medium fractal category.

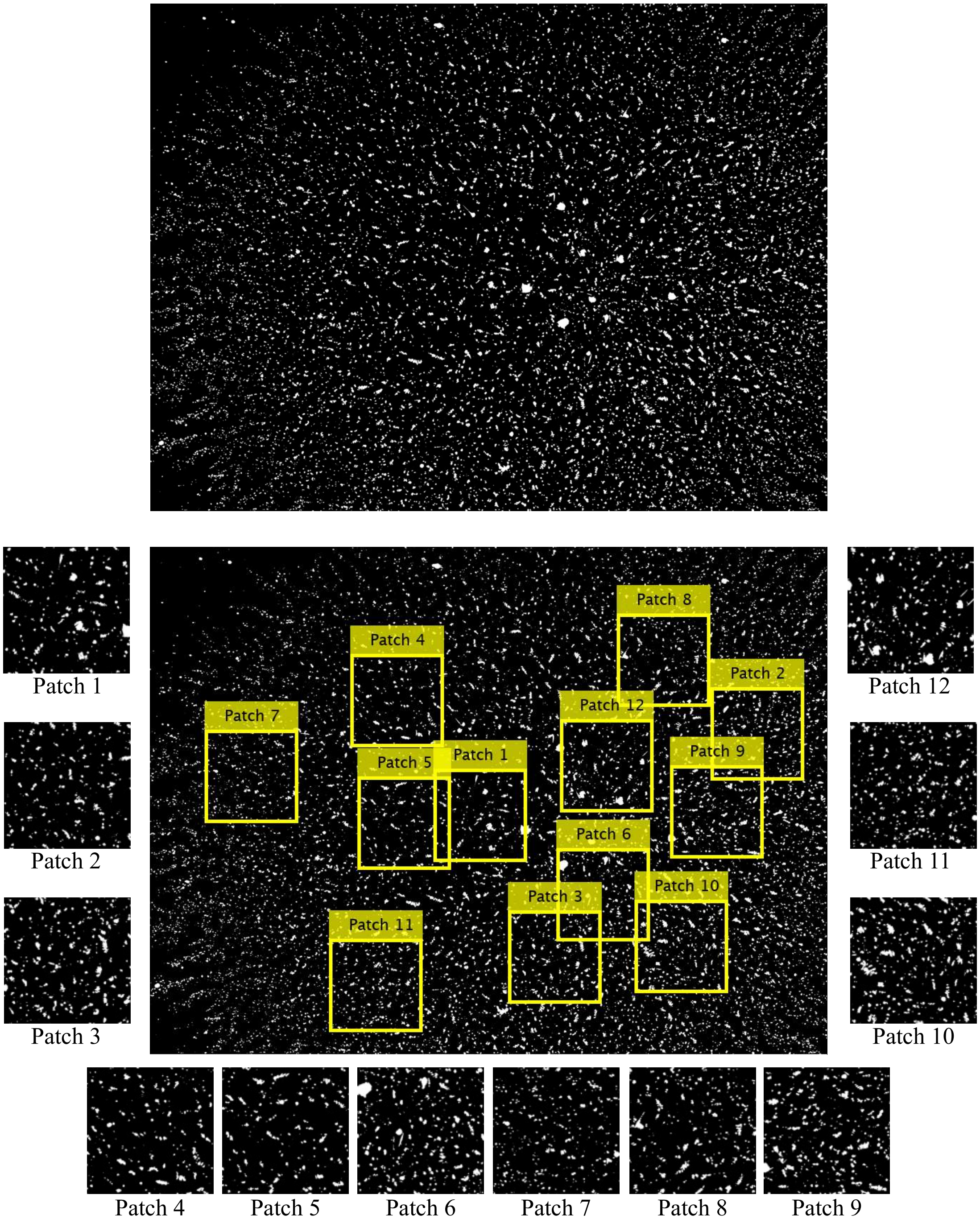

Figure S 6. T mixing procedure image and its spatial location of patches. The image with the highest texture composition in the less fractal category is shown at the top. The twelve patches, which correspond to the less fractal category, are shown at the bottom.

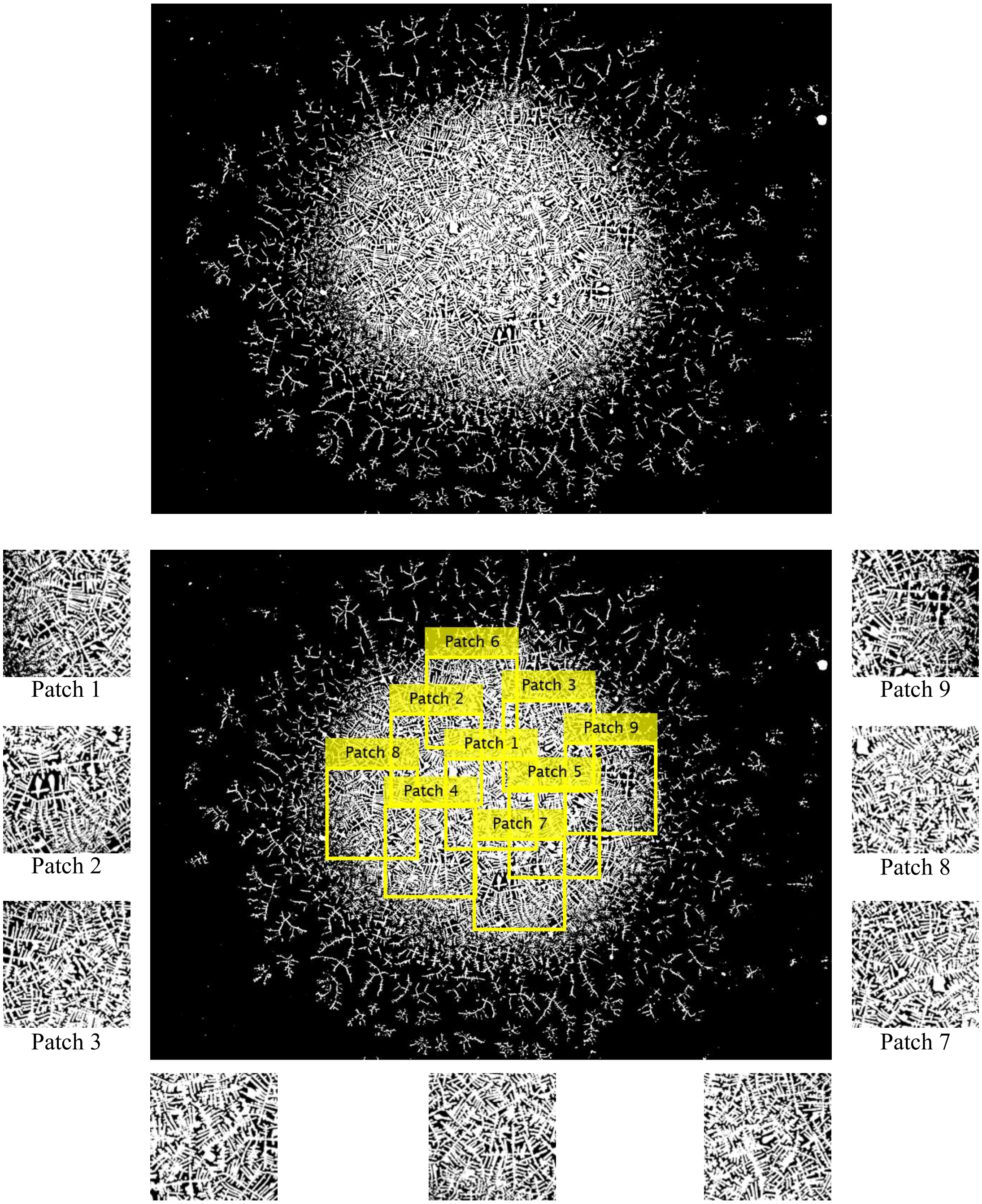

Figure S 7. L mixing procedure image and its spatial location of patches. The image assigned with the more fractal category is shown at the top. Its nine patches can be observed at the bottom of the figure.
